# Effect of antecedent moderate-intensity exercise on the glycemia-increasing effect of a 30-sec maximal sprint: a sex comparison

**DOI:** 10.14814/phy2.12386

**Published:** 2015-05-27

**Authors:** Tara D Justice, Greta L Hammer, Raymond J Davey, Nirubasini Paramalingam, Kym J Guelfi, Lynley Lewis, Elizabeth A Davis, Timothy W Jones, Paul A Fournier

**Affiliations:** 1 School of Sport Science, Exercise, and Health, The University of Western AustraliaCrawley, Western Australia, Australia; 2 Telethon Kids Institute, The University of Western AustraliaCrawley, Western Australia, Australia; 3 School of Paediatrics and Child Health, The University of Western AustraliaPerth, Western Australia, Australia; 4 Department of Endocrinology and Diabetes, Princess Margaret HospitalPerth, Western Australia, Australia; 5 Department of Medicine, Christchurch Heart Institute, The University of OtagoDunedin, New Zealand

**Keywords:** Blood glucose, counterregulation, glucoregulation, high-intensity exercise

## Abstract

This study investigated whether a prior bout of moderate-intensity exercise attenuates the glycemia-increasing effect of a maximal 30-sec sprint. A secondary aim was to determine whether the effect of antecedent exercise on the glucoregulatory response to sprinting is affected by sex. Participants (men *n* = 8; women *n* = 7) were tested on two occasions during which they either rested (CON) or cycled for 60-min at a moderate intensity of ~65% 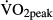 (EX) before performing a 30-sec maximal cycling effort 195 min later. In response to the sprint, blood glucose increased to a similar extent between EX and CON trials, peaking at 10 min of recovery, with no difference between sexes (*P* > 0.05). Blood glucose then declined at a faster rate in EX, and this was associated with a glucose rate of disappearance (*R*_d_) that exceeded the glucose rate of appearance (*R*_a_) earlier in EX compared with CON, although the overall glucose *R*_a_ and *R*_d_ profile was higher in men compared with women (*P* < 0.05). The response of growth hormone was attenuated during recovery from EX compared with CON (*P* < 0.05), with a lower absolute response in women compared with men (*P* < 0.05). The response of epinephrine and norepinephrine was also lower in women compared with men (*P* < 0.05) but similar between trials. In summary, a prior bout of moderate-intensity exercise does not affect the magnitude of the glycemia-increasing response to a 30-sec sprint; however, the subsequent decline in blood glucose is more rapid. This blood glucose response is similar between men and women, despite less pronounced changes in glucose *R*_a_ and *R*_d_, and a lower response of plasma catecholamines and growth hormone to sprinting in women.

## Introduction

During low-to-moderate intensity exercise, several regulatory mechanisms interact to maintain blood glucose concentrations within a narrow physiological range (Coggan 1991). This is essential for maintaining an adequate supply of glucose to the brain despite the increased metabolic demands of the working muscles during physical exercise. However, this precise homeostatic balance between glucose production and utilization is disrupted in response to high-intensity exercise (>80% 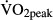), with blood glucose concentrations increasing above resting levels (Purdon et al. 1993). The glycemia-raising effect of high-intensity exercise has been attributed to a sympathoadrenal-mediated increase in the rate of glucose appearance in the circulation that far exceeds the increase in the rate of glucose utilization by active muscles (Marliss and Vranic 2002).

Recently, we have shown that as little as 10-sec of maximal exercise is capable of causing an increase in blood glucose concentrations in both healthy individuals and those with type 1 diabetes (Fahey et al. 2012; Davey et al. 2014). We have also found that performing one or several short sprints is protective against hypoglycemia in individuals with type 1 diabetes (Guelfi et al. 2005, 2007a,b; Bussau et al. 2006, 2007). However, the factors affecting the glycemia-raising effect of sprinting are poorly understood. This is an important issue to address before advocating the use of ‘sprinting’ as a means to reduce the risk of hypoglycemia in physically active individuals with type 1 diabetes. Since antecedent moderate-intensity exercise has previously been shown to reduce the glucoregulatory responses to subsequent moderate-intensity exercise performed hours later on the same day in nondiabetic individuals (Galassetti et al. 2001), it is possible that the glucoregulatory and glycemia-raising responses to a short sprint may also be diminished if the sprint is preceded by a bout of exercise. Therefore, the primary purpose of this study was to examine whether antecedent moderate-intensity exercise reduces the glycemia-increasing effect of a 30-sec maximal sprint performed several hours later. Furthermore, previous studies have shown marked differences in the glucoregulatory responses to moderate-intensity exercise between the sexes (Davis et al. 2000; Galassetti et al. 2001, 2002, 2004). For instance, women have been reported to experience a lower absolute increase in plasma catecholamine concentrations and a lesser suppression of insulin secretion in response to prolonged submaximal exercise compared with men (Davis et al. 2000). Yet, the blunting of the glucoregulatory responses to moderate-intensity exercise following antecedent hypoglycemia (Galassetti et al. 2004) and antecedent moderate exercise (Galassetti et al. 2001) has been shown to be more pronounced in men compared with women. Accordingly, the secondary aim of this study was to determine whether the effects of antecedent exercise on the responses to a short sprint is influenced by sex. Before addressing these issues in individuals with type 1 diabetes, we performed this study in nondiabetic participants to understand how antecedent exercise affects the glycemic response to sprinting under nonpathological conditions.

## Methods

### Participants

Fifteen healthy physically active men (*n* = 8) and women (*n* = 7) provided informed consent to participate (Table1). Exclusion criteria were exercise-limiting musculoskeletal injuries or taking any form of medication. Ethics approval was granted by both the Princess Margaret Hospital (PMH) and The University of Western Australia Human Research Ethics Committee.

**Table 1:** Participant characteristics (mean ± SD).

	Men (*n* = 8)	Women (*n* = 7)
Age (year)	22 ± 2	21 ± 1
Body Mass (kg)	75.6 ± 10.4	63.9 ± 10.7
Height (cm)	177.6 ± 7.7	166.2 ± 9.9^1^
BMI (kg·m^−2^)	23.9 ± 2.2	23.0 ± 2.1
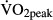 (mL·kg·min^−1^)	48.6 ± 6.6	44.4 ± 4.3

1 Indicates significant difference from men (*P* < 0.05).

### Experimental design

Each participant was required to attend the Clinical and Metabolic Research Unit at PMH on three occasions, each separated by at least 1 week. The first visit was a familiarization session, with the subsequent two visits involving an exercise trial (EX) and a resting control trial (CON) administered in a counterbalanced order to determine the effect of an antecedent bout of exercise on the glucose and counterregulatory responses to a maximal sprint effort. For EX, participants performed 60 min of moderate-intensity exercise and then rested for 3 h and 15 min before performing a 30-sec maximal sprint effort. For CON, participants rested for 60 min prior to performing the 30-sec maximal sprint 3 h and 15 min later. A 30-sec sprint was adopted because of the more pronounced rise in blood glucose levels expected with a 30-sec sprint compared with a 10-sec sprint. For women, testing was performed during the follicular phase of the menstrual cycle (days 3–11), with ~28 days between trials.

### Familiarization session

Participants were instructed to avoid caffeine, alcohol, and physical activity in the 24 h prior to arriving at the laboratory. Standing height (Model 240; SECA, Hamburg, Germany) and body mass were measured (Model 770; SECA Alpha) before an incremental exercise test was performed on a Front Access Cycle Ergometer (Exertech; Repco, Melbourne, Vic., Australia) to establish each participant's peak rate of oxygen consumption (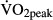; VMax Spectra; SensorMedics Corporation, Yorba Linda, CA). Briefly, this involved measuring resting oxygen consumption for 3 min, followed by cycling at 50–90 W, depending on the participant's current level of physical activity. The intensity of cycling was increased by 40 W every 3 min until volitional exhaustion was reached. During exercise, participants breathed through a calorimetry mask so that the volume of expired gas, as well as the fractions of oxygen and carbon dioxide in the expired air, could be measured using a computerized gas analysis system (VMax Spectra; SensorMedics Corporation). These parameters were used to calculate each participant's 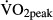, which was subsequently used to determine the appropriate workload (65% 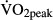) for the EX trial.

### Experimental trials

Three days prior to each experimental trial, each participant was fitted with an accelerometer (Activity Monitor GT1M; Actigraph, Pensacola, FL) to monitor physical activity since antecedent exercise has been shown to blunt the glucoregulatory responses to subsequent exercise (Galassetti et al. 2001). Participants were also asked to record their food and drink intake and to abstain from caffeine and alcohol for 24 h prior to each trial. Participants were required to consume the same food and drink on the day prior to the subsequent trial to standardize energy intake.

At 0720 h on the morning of each trial, participants consumed a liquid breakfast (16 kJ·kg^−1^ body mass; Sanitarium Up & Go Liquid Breakfast, Sanitarium, Berkeley Vale, NSW, Australia; 61% carbohydrate, 19% fat, 20% protein) following a 10-h overnight fast. Upon arrival to the laboratory at 0800 h, the participant began either cycling on the ergometer for 60 min at 65% 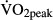 (EX) or sat resting on the ergometer for 60 min (CON). This exercise intensity and duration was employed to reflect ‘real world’ practices and current exercise guidelines (American College of Sports Medicine 2011). During the 60 min of antecedent exercise or rest, expired air was analyzed (VMax Spectra; SensorMedics Corporation) to confirm the prescribed exercise intensity. In addition, blood lactate was sampled from the fingertip at 15 and 45 min of EX or CON (Lactate Pro, Arkray Inc., Kyoto, Japan).

Immediately after this 60-min period of either exercise or rest, a 20-gauge cannula (BD Insyte^™^; Becton Dickinson, Sandy, UT) was inserted into an antecubital vein for blood sampling and kept patent with regular flushing of 0.9% (w/v) saline. A blood sample was taken to measure the background enrichment of deuterated glucose before inserting another cannula into the contralateral arm for the infusion of the nonradioactive stable isotope [6,6-^2^H]glucose (ASENA Syringe Driver, Alaris Medical System, Basingstoke, UK) to measure the rate of glucose appearance (*R*_a_) and disappearance (*R*_d_). This infusion was commenced 30 min after the cessation of the 60 min period of exercise or rest with an initial priming bolus of 3 mg·kg^−1^ body mass, followed by a constant infusion of 2.4 mg·kg^−1^·h^−1^ for the remainder of the testing period as we have previously described (Guelfi et al. 2007a; Fahey et al. 2012; Davey et al. 2014). Participants were required to rest for a further 150 min for isotopic equilibrium to be reached prior to the sprint.

The 30-sec maximal sprint was commenced after a 3-sec countdown with both feet strapped into the ergometer pedals and the dominant leg raised to a 45° angle, ready to push down. Participants were told to cycle as hard as they could from the start of the sprint, and not to pace themselves. Verbal encouragement was provided. Following the sprint, each participant dismounted the bike and rested for 60 min for the response of glucose kinetics and glucoregulatory hormones to be monitored. Venous blood samples (23 mL) were taken 15 and 5 min before the sprint, as well as at 0, 5, 10, 15, 30, 45, and 60 min of recovery. For each sample, the participant's forearm was warmed in a hot box (Omega CN 370, Sydney, Australia) at 55–60°C to arterialize the venous blood.

### Measurement of blood metabolites and hormones

Blood glucose and lactate concentrations were determined using a YSI Analyser (YSI Life Sciences, Yellow Springs, OH). The remaining blood was aliquotted to appropriate collection tubes prior to centrifugation at 4°C at 3500 rpm (Eppendorf Centrifuge 5810 R, Hamburg, Germany), with the resultant serum or plasma stored at −40°C (with the exception of the deuterated glucose and catecholamines which were stored at −80°C). Plasma collected in a lithium heparin tube was assayed for insulin by noncompetitive chemiluminescent immunoassay (Abbott Architect i2000, Abbott Park, IL). Plasma was collected for the assay of pancreatic-specific glucagon in a K-EDTA tube with a Benzamidine HCL additive, and subsequently assayed using a noncompetitive radio-immunoassay (Siemens Medical Solutions Diagnostics Ltd, Pleasanton, CA). Growth hormone was assayed from serum by a noncompetitive enzyme immunoassay with a chemiluminescent substrate (Siemens Immulite 2000 XPi; Siemens Medical Solutions Diagnostics Ltd). Serum was also collected for the assay of cortisol using a competitive chemiluminescent immunoassay (Abbott Architect i2000). Epinephrine and norepinephrine were collected in a lithium heparin tube with a sodium metabisulfite additive before being extracted on alumina and eluted with acetic acid. The extracted catecholamines were separated using reverse phase HPLC (Ultrasphere, Beckman Coulter, Albany North Shore City, New Zealand) using a Shimadzu HPLC system (Shidmadzu Scientific Instruments, Sydney, Australia) with an ESA Coulochem-II Electrochemical Detector (Chelmsford, MA).

The level of plasma enrichment of [6,6-^2^H]glucose was determined from plasma collected in a standard lithium heparin tube using gas chromatography mass spectrometry (GCMS; Agilent Selective Detector, Agilent technologies, Ryde, NSW, Australia). This method, as previously described by Hannestad and Lundblad (1997), involved the conversion of [6,6-^2^H]glucose to its aldonitrile derivative, which was subsequently measured using GCMS. Glucose *R*_a_ and *R*_d_ were then calculated using the one-compartment fixed-volume nonsteady state model of Steele (Wolfe and Chinkes 2005). This model assumes that the [6,6-^2^H]glucose distribution volume is stable, and that changes in plasma glucose concentration only result from changes in glucose *R*_a_ and *R*_d_.

### Statistical analyses

Mixed model analysis of variance with repeated measures (time and trial) and between group comparisons for sex were used to determine whether differences existed between trials, over time or between sexes with post hoc comparisons to locate differences as appropriate (SPSS 19.0 Software for Windows, Chicago, IL). In addition, the area under the curve (AUC) was calculated using the trapezoidal rule for the blood glucose and hormonal responses to the sprint and compared between conditions and sexes using repeated measured repeated measures ANOVA. Pearson correlation was undertaken between relative mean power output in the 30-sec sprint and the maximum change in blood glucose concentration. Statistical significance was accepted at *P* < 0.05. With the exception of all figures (mean ± SEM), all data are expressed as mean ± SD.

## Results

### Characteristics of the 60-min antecedent bout of exercise and 30-sec maximal sprint effort

In the 24 h preceding each trial, there was no difference in the total number of steps taken as determined from accelerometry (*P* = 0.204), suggesting that prior activity levels were matched between trials. During the 60 min of antecedent exercise (EX), participants worked at 62 ± 8% of 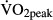, compared with the equivalent rest period (CON) in which oxygen consumption remained at basal levels (10 ± 2% 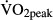), with no difference between men and women (*P* > 0.05). Antecedent exercise was accompanied by an elevation in both heart rate (150 ± 12 bpm) and blood lactate concentrations (3.2 ± 1.4 mmol·L^−1^) compared with antecedent rest (79 ± 6 bpm; 1.8 ± 0.7 mmol·L^−1^; *P* < 0.05).

During the subsequent 30-sec sprint effort, mean power output (CON 667 ± 214 W; EX 671 ± 228 W) and peak power (CON 1052 ± 403 W; EX 1085 ± 431 W) were similar between trials, although absolute power output was higher in men compared with women (*P* < 0.05). The sprint was associated with a significant rise in heart rate, which peaked immediately postsprint (CON 155 ± 16 bpm; EX 159 ± 15 bpm) and returned to baseline levels by 30 min of recovery. Likewise, blood lactate concentration increased in response to the sprint in both trials, reaching a similar peak at 5 min of recovery (CON 8.7 ± 1.7 mmol·L^−1^; EX 8.6 ± 1.8 mmol·L^−1^; *P* = 0.304) before progressively returning to baseline. The blood lactate response to the sprint was higher in men compared with women (men 9.6 ± 1.8 mmol·L^−1^; women 7.7 ± 1.1 mmol·L^−1^; *P* < 0.05).

### Blood glucose and insulin response to the 30-sec maximal sprint

**Figure 1:**
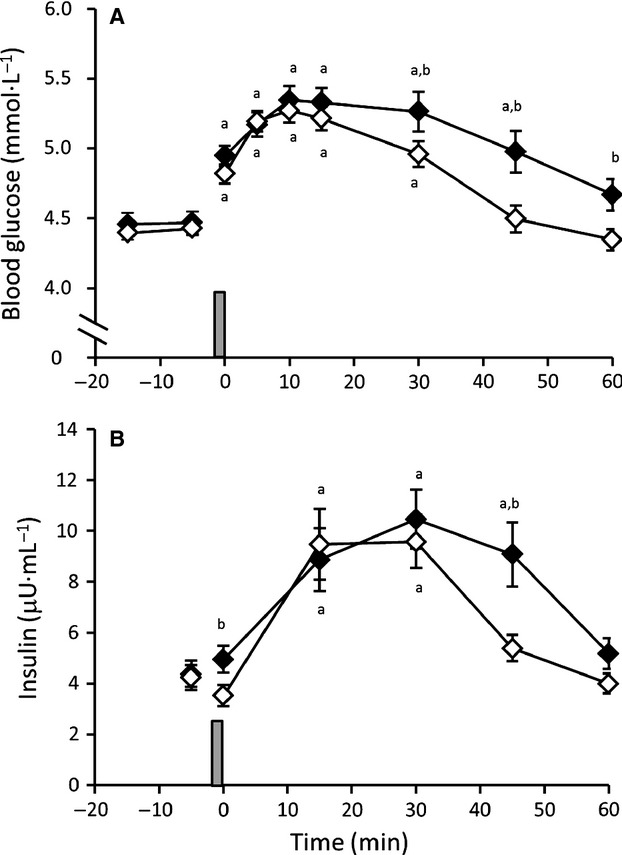
Response of (A) blood glucose and (B) plasma insulin to a 30-sec maximal sprint (represented by vertical bar) performed after 60 min of antecedent exercise (◊ EX) or rest (♦ CON) (*n* = 15; mean ± SEM). ^a^Significant difference from baseline (time -5 min); ^b^Significant difference between CON and EX (*P* < 0.05).

At baseline, the blood glucose concentration was similar between trials and sexes (Men CON 4.46 ± 0.20 mmol·L^−1^; EX 4.44 ± 0.15 mmol·L^−1^; Women CON 4.48 ± 0.44 mmol·L^−1^; EX 4.41 ± 0.23 mmol·L^−1^; *P* = 0.907). In response to the 30-sec maximal sprint, there was an interaction effect of trial and time (*P* = 0.032), but no effect of sex (*P* = 0.337). For this reason, the blood glucose response of men and women is presented together (Fig.1A). Blood glucose levels increased significantly and similarly in both trials in response to the sprint, reaching a peak at 10 min of recovery (Men CON 5.44 ± 0.38 mmol·L^−1^; EX 5.43 ± 0.23 mmol·L^−1^; Women: CON 5.21 ± 0.41 mmol·L^−1^; EX 5.09 ± 0.38 mmol·L^−1^; *P* = 0.405). After this time, blood glucose concentrations began to decline, returning to baseline by 45 min of recovery in EX (*P* = 0.447) and by 60 min of recovery in CON (*P* = 0.135). The decline in blood glucose concentrations was more rapid in EX, resulting in significantly lower blood glucose concentrations from 30 to 60 min of recovery compared with CON (*P* < 0.05). The more rapid decline in blood glucose concentration in EX was reflected, in part, by a tendency for lower overall glucose AUC in EX (292 ± 18 mmol·L^−1^ over 60 min) compared with CON (307 ± 28 mmol·L^−1^ over 60 min; *P* = 0.054), with no main effect of sex (*P* = 0.289). The relationship between relative mean power output and the maximum change in blood glucose concentration approached significance (*r* = 0.528, *P* = 0.052).

The response of plasma insulin concentrations to the 30-sec maximal sprint was also similar between sexes (*P* = 0.347) and for this reason, the combined results of men and women are presented (Fig.1B). Plasma insulin was similar between trials prior to the 30-sec sprint, but immediately after sprinting the plasma insulin concentration was lower in EX compared with CON (*P* < 0.001), after which time insulin began to rise in both trials, reaching a similar peak at 30 min of recovery. Plasma insulin returned to baseline at 45 min of recovery in EX, and at 60 min of recovery in CON. Insulin was higher at 45 min of recovery in CON compared to EX (*P* = 0.016).

### Glucose rate of appearance and disappearance in response to the 30-sec maximal sprint

**Figure 2:**
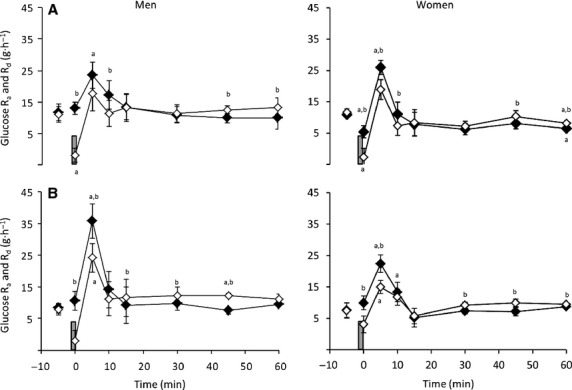
Glucose rate of appearance (♦) and disappearance (◊) in response to a 30-sec maximal sprint (represented by vertical bar) performed after (A) 60 min of rest or (B) after 60 min of antecedent exercise in men (*n* = 8) and women (*n* = 7) (mean ± SEM). ^a^Significant difference from baseline (time -5 min); ^b^Significant difference between *R*_a_ and *R*_d_ (*P* < 0.05).

The response of glucose *R*_a_ and *R*_d_ to the 30-sec maximal sprint performed in EX and CON in men and women is displayed in Fig.2A and B. Despite a similar blood glucose response to the 30-sec sprint between sexes, there was a significant effect of sex on glucose *R*_a_ and *R*_d_, with a higher overall glucose *R*_a_ and *R*_d_ response in men compared with women for both CON (*P* = 0.006) and EX trials (*P* = 0.042). In addition to this main overall effect of sex, post hoc analyses revealed that glucose *R*_a_ was higher in men compared with women specifically at 0 min of recovery in CON (*P* = 0.018) and approached significance at 5 min of recovery in EX (*P* = 0.059). However, the pattern of change in glucose *R*_a_ and *R*_d_ was similar in men and women, with a significant increase in glucose *R*_a_ above baseline at 5 min postsprint in both CON and EX (*P* < 0.05), before returning to baseline levels at 10–15 min postsprint where glucose *R*_a_ remained for the duration of recovery (except for a drop below baseline in women in the CON trial at 60 min postsprint; *P* = 0.018). In contrast, glucose *R*_d_ declined immediately following the 30-sec sprint in both trials, dropping significantly below baseline in CON for both men and women. After this initial decline, glucose *R*_d_ increased (reaching significance at 5 min of recovery in women in the EX trial) before quickly returning to preexercise levels.

Within each trial, glucose *R*_a_ was above glucose *R*_d_ immediately following the 30-sec sprint in both men and women. Glucose *R*_a_ was greater than glucose *R*_d_ until 15 min of recovery in CON and 10 min of recovery in EX. Following this, glucose *R*_a_ was matched by glucose *R*_d_ until 45 min of recovery in CON, compared with 15 min (men) and 30 min (women) of recovery in EX. At these times, glucose *R*_d_ was above glucose *R*_a_ for the majority of the remaining recovery period (*P* < 0.05).

### Glucoregulatory hormone response to the 30-sec maximal sprint

**Figure 3:**
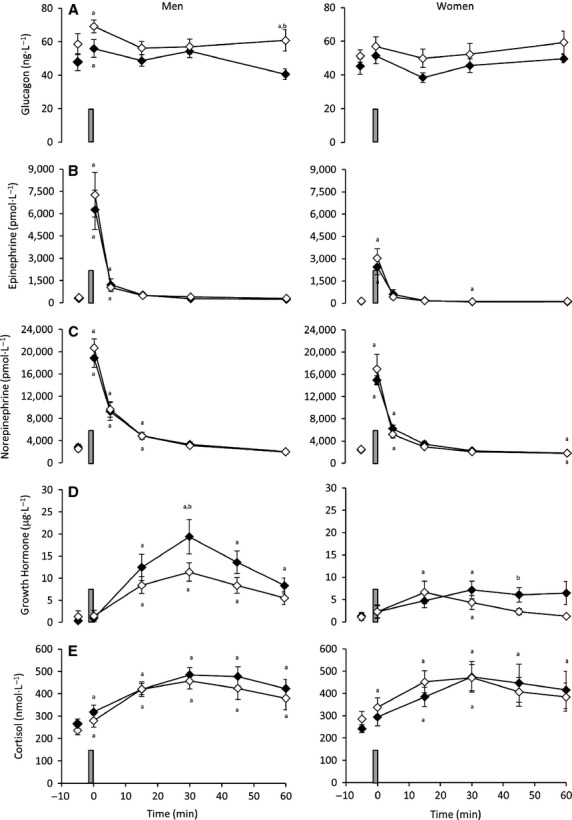
Response of (A) glucagon (B) epinephrine (C) norepinephrine (D) growth hormone and (E) cortisol to a 30-sec maximal sprint (represented by vertical bar) performed after 60 min of antecedent exercise (◊ EX) or rest (♦ CON) in men (*n* = 8) and women (*n* = 7) (mean ± SEM). ^a^Significant difference from baseline (time -5 min); ^b^Significant difference between CON and EX (*P* < 0.05).

Plasma glucagon was similar between trials prior to the 30-sec sprint (*P* > 0.05; Fig.3A). There was no main effect of sex on the response of plasma glucagon concentrations to the 30-sec maximal sprint (*P* = 0.238); however, glucagon transiently increased immediately postsprint and was 60 min higher following the sprint in EX compared with CON in men (*P* = 0.015). Accordingly, the overall AUC for glucagon between trials approached significance (*P* = 0.058), with a tendency for higher values in EX compared with CON, but no difference between sexes (*P* = 0.415). Plasma epinephrine (Fig.3B) and norepinephrine concentrations (Fig.3C) were similar at baseline between trials (*P* > 0.05). A significant increase in catecholamine concentration was noted following the sprint in both EX and CON (*P* < 0.05), with no difference between trials. The overall AUC for epinephrine was significantly higher in EX compared with CON (*P* = 0.010). An effect of sex was also noted for both epinephrine (*P* = 0.01) and norepinephrine (*P* = 0.006), with a lower absolute increase in women compared with men. AUC analysis supported these results, with lower overall AUC for both epinephrine (*P* = 0.017) and norepinephrine (*P* = 0.004) in women compared with men. With respect to growth hormone, a rise was noted following the sprint, with peak concentrations reached at 30 min of recovery (Fig.3D). This growth hormone response was blunted in EX and affected by sex, with an attenuated rise in women (*P* = 0.045). Between trials, growth hormone was lower at 30 min of recovery in men (*P* = 0.049) and 45 min of recovery in women (*P* = 0.048) in the EX trial compared with CON. Overall AUC for growth hormone mirrored these results, with lower values in EX compared with CON (*P* = 0.034) and women compared with men (*P* = 0.022). Plasma cortisol increased in response to the sprint (Fig.3E). This increase was similar between trials (*P* = 0.712) and sexes (*P* = 0.820), with peak cortisol concentrations observed 30 min postsprint. AUC analysis supported this, with no difference between trials (*P* = 0.69) or sexes (*P* = 0.974).

## Discussion

This study shows that 60 min of moderate-intensity exercise does not affect the magnitude of the rise in blood glucose concentration in response to a 30-sec maximal sprint performed approximately 3 h later; however, the subsequent decline in blood glucose concentration is more rapid following antecedent exercise. Furthermore, antecedent exercise does not attenuate the glucoregulatory responses to sprinting, with the exception of a lesser rise in growth hormone concentration. With respect to the influence of sex, the blood glucose response to sprinting following antecedent exercise is similar between men and women, despite less pronounced changes in the rates of glucose appearance and disappearance, and a lower response of the catecholamines and growth hormone to sprinting in women.

The observation that antecedent exercise did not affect the magnitude of the rise in glycemia and had little effect on the responses of most glucoregulatory hormones to sprinting is in contrast with the finding that antecedent exercise reduces the glucoregulatory response to subsequent moderate-intensity exercise (Galassetti et al. 2001). In particular, moderate-intensity exercise has been shown to attenuate the responses of plasma epinephrine, norepinephrine, growth hormone, glucagon, and cortisol to a second bout of moderate-intensity exercise performed several hours later (Galassetti et al. 2001). In the present study, antecedent exercise did not affect the responses of epinephrine, norepinephrine, glucagon, or cortisol to sprinting, but growth hormone concentration increased to a lesser extent. This difference is likely related to the nature of the exercise stimulus, with the maximal ‘all-out’ sprint perhaps overriding any suppression caused by antecedent exercise. In support of this notion, the increases in plasma epinephrine and norepinephrine concentrations in response to sprinting were much more pronounced compared with the responses to moderate-intensity exercise (Galassetti et al. 2001).

The similar blood glucose response to sprinting following antecedent exercise between men and women was unexpected considering that previous studies have shown marked differences in the glucoregulatory responses to moderate-intensity exercise between sexes (Davis et al. 2000; Galassetti et al. 2001, 2002, 2004). In particular, these studies have shown that, compared with men, women produce lower counterregulatory responses to moderate-intensity exercise (Davis et al. 2000; Galassetti et al. 2002), but these responses are less attenuated following antecedent hypoglycemia (Galassetti et al. 2004) and antecedent exercise (Galassetti et al. 2001). Our findings support the observation that women have less pronounced counterrregulatory responses to exercise based on the lower growth hormone and catecholamine responses to sprinting compared with men. However, the effect of antecedent exercise on most of these glucoregulatory responses to sprinting does not appear to be influenced by gender (i.e., no lesser attenuation of the response in women) given the similar responses between EX and CON trials. The reason for this similar effect of antecedent exercise on the glucoregulatory responses to sprinting in men and women is unclear, but may again be related to the nature of the exercise stimulus, with the maximal ‘all-out’ sprint perhaps overriding the potential for any suppression caused by antecedent exercise.

With respect to the mechanisms whereby glycemia increases in response to a 30-sec sprint, these appear similar to those associated with a 10-sec sprint, but markedly different from that observed in response to intense aerobic exercise. The increase in blood glucose concentration at the onset of recovery from the 30-sec sprint was associated with a rapid transient fall in glucose *R*_d_, while glucose *R*_a_ remained near preexercise levels. Over the following 5–10 min of recovery, the further rise in blood glucose took place despite a marked increase in glucose *R*_d_ in both trials. The increase in blood glucose concentration is likely the result of the increase in glucose *R*_a_ exceeding the rise in glucose *R*_d_, with the pattern of *R*_a_ and *R*_d_ responses being similar between men and women. It is noteworthy that the transient fall in glucose *R*_d_ and the lack of increase in glucose *R*_a_ at the onset of recovery have also been observed in response to a 10-sec maximal sprint in individuals with and without type 1 diabetes (Fahey et al. 2012). However, this pattern of change in glucose *R*_a_ and *R*_d_ differs from that associated with sustained high-intensity aerobic exercise, where glucose *R*_a_ and *R*_d_ reach near maximal levels at the onset of recovery before declining as recovery progresses (Purdon et al. 1993; Sigal et al. 1994; Marliss et al. 2000; Marliss and Vranic 2002). Such observations suggest that the mechanisms underlying the glycemia-increasing effect of intense exercise are highly dependent on the specific intensity and duration of the exercise bout.

The large rise in plasma catecholamines immediately after sprinting suggests that these hormones may play some role in mediating the increase in glycemia seen 5–10 min following the sprint. Others have also reported that the rise in blood glucose levels associated with high-intensity exercise is accompanied by an immediate increase in plasma epinephrine and norepinephrine (Purdon et al. 1993; Sigal et al. 1994; Marliss et al. 2000). This increase in catecholamines may contribute to the rise in blood glucose via inhibition of glucose *R*_d_ given the evidence that the catecholamines inhibit muscle glucose uptake during exercise (Howlett et al. 1999; Watt and Hargreaves 2002), although it is important to note that others have shown that catecholamine infusion during exercise enhances rather than inhibits glucose *R*_d_ (Kreisman et al. 2000, 2001). The increase in catecholamines may also stimulate glucose *R*_a_, with both epinephrine and norepinephrine associated with increased rates of hepatic glucose production (Rizza et al. 1979; Purdon et al. 1993; Kreisman et al. 2000, 2001, 2003). Similarly, the higher overall glucose *R*_a_ in response to sprinting in men compared with women may be explained on the grounds of a more pronounced increase in catecholamine concentrations in men. However, these proposed roles for catecholamines must be taken with caution given that the rise in catecholamine concentrations and glucose *R*_a_ do not precisely coincide. Interestingly, despite the lower overall glucose *R*_a_ in the women, the absolute rise in blood glucose concentration was equivalent between sexes. The most likely explanation for this is that the rise in glucose *R*_a_ in both the men and women was accompanied by a proportionate rise in glucose *R*_d_.

Later in recovery, the more rapid decline in blood glucose concentrations in the EX trial compared with CON is likely the result of glucose *R*_d_ exceeding glucose *R*_a_ at an earlier stage. Indeed, glucose *R*_d_ was significantly higher than glucose *R*_a_ in the EX trial from 15 to 45 min of recovery in men and from 30 to 60 min of recovery in women, while in the CON trial glucose *R*_d_ did not exceed glucose *R*_a_ until 45 min of recovery in both men and women. The more rapid decline in blood glucose concentration and more pronounced mismatch between glucose *R*_a_ and *R*_d_ in EX compared with CON might be explained by the lower level of growth hormone during mid-to-late recovery in EX in both men and women, since elevated growth hormone can acutely inhibit glucose uptake (Møller et al. 1990, 1992). The reason for this attenuated growth hormone response is not clear, although Galassetti et al. (2001) also observed an attenuated growth hormone response to a second bout of moderate exercise performed several hours later. The more rapid decline in blood glucose in EX compared with CON was also associated with lower plasma insulin levels in EX at 45 min of recovery. The similar glucose *R*_d_ in both trials despite lower plasma insulin levels in EX is consistent with greater insulin sensitivity. Since the level of muscle glycogen is an important determinant of insulin sensitivity (Derave et al. 2000), any increase in insulin sensitivity in EX may be the result of lower postsprint muscle glycogen levels due to the expected cumulative effect of antecedent exercise and sprinting on muscle glycogen stores.

In summary, we have found that an antecedent bout of moderate-intensity exercise does not affect the magnitude of the rise in blood glucose concentration in response to a 30-sec sprint performed hours later; however, it does cause blood glucose levels to decline more rapidly later during recovery. This faster rate of decline in blood glucose concentration during recovery from a sprint performed after a bout of antecedent exercise is associated with a blunted growth hormone response to sprinting. These findings raise the question of whether antecedent exercise performed in the hours before sprinting may also impair the effectiveness of sprinting for raising glycemia and decreasing the risk of hypoglycemia in individuals with type 1 diabetes. For this reason, future research should examine the extent to which antecedent exercise, as well as other factors, affects the glucoregulatory benefits of ‘sprinting’, before advocating the use of sprinting as a means of reducing the risk of exercise-mediated hypoglycemia in individuals with type 1 diabetes. Finally, the counterregulatory and glucose *R*_a_ and *R*_d_ responses to sprinting are reduced in women compared with men; however, the blood glucose response to sprinting is equivalent since the pattern of these responses is similar between sexes.

## Conflicts of Interest

No conflict of interest to declare.
